# Examining social competence, self-perception, quality of life, and internalizing and externalizing symptoms in adolescent females with and without autism spectrum disorder: a quantitative design including between-groups and correlational analyses

**DOI:** 10.1186/s13229-015-0044-x

**Published:** 2015-09-17

**Authors:** T. Rene Jamison, Jessica Oeth Schuttler

**Affiliations:** Center for Child Health and Development, University of Kansas Medical Center, 3901 Rainbow Boulevard, Kansas, KS 66160 USA

**Keywords:** Autism, Females with autism, Adolescents, Social competence, Self-perception, Internalizing disorders

## Abstract

**Background:**

Adolescent females with an autism spectrum disorder (ASD) are an understudied population, yet are also quite vulnerable, due to the increased complexities of social interaction and increased risk for internalizing symptoms in adolescence. Most research literature currently focuses on males with ASD, limiting our understanding of social experiences for females with ASD, and thus the potential to better inform supports and intervention to promote social-emotional functioning. This study examined similarities and differences in selected indicators of social-emotional health (social competence, self-perception, quality of life) and problematic behaviors such as externalizing and internalizing symptoms for adolescent females with and without ASD.

**Methods:**

This study employed a quantitative design utilizing correlational analysis as well as *t* test comparisons to examine selected indicators of social-emotional health and problematic symptoms using the Social Skills Improvement System (SSIS), Youth Quality of Life Instrument (YQOL), and the Self-Perceptions Profile for Adolescents (SPPA) for adolescent females with ASD in relation to their typically developing peers.

**Results:**

Significant differences were found between females with and without ASD in terms of their self-ratings of social-emotional health and problematic behaviors. The no-ASD group rated themselves higher across all areas of social-emotional health. Findings also suggest strong relationships between these constructs, especially for females without ASD. Parent reports of autism symptoms and social-emotional health indicated that as symptoms of autism are more severe, so too was the impact on individuals’ social competence.

**Conclusions:**

Adolescent females with ASD perceive themselves as having lower social competence, self-worth, and quality of life and higher levels of internalizing and externalizing symptoms as compared to their typically developing peers. Parent ratings indicate that higher levels of autism symptoms relate to lower levels of social competence. These findings lend support to the postulate that adolescent females with ASD are more vulnerable than their typically developing counterparts due to the compounded impact of ASD symptoms on social-emotional health and the higher risk for internalizing disorders for adolescent girls. Limitations and implications for further research and intervention are discussed.

## Background

Social impairment is a core deficit for the diagnosis of autism spectrum disorder (ASD) [1], impacting individuals’ relationships across all domains. Social impairment compounds into adolescence and beyond due to the increasing complexity of age-typical interactions [2]. Adolescence is a time of social vulnerability and biological change for typically developing individuals, even more so for those with disabilities such as ASD that significantly impact social functioning as well as adaptive skills such as self-care. Females represent 1 in 5 individuals with ASD, yet adolescent females may experience the most significant impact of social impairment on their functioning [3, 4]. The confluence of difficulties with social interaction and self-care have the potential to negatively influence self-perceptions, leading to an increased risk for internalizing disorders for this population above and beyond their neurotypical peers [4]. There is ample literature describing the variability of how ASD impacts males with very little information about how ASD is expressed in females and no published intervention research specifically targeted to adolescent females with ASD [3].

### Social competence and self-perception

Interpersonal skills are a keystone behavior in developing healthy relationships and in successful adjustment. Impairments increase risks for internalizing and externalizing problems such as disruptive behaviors, anxiety, and depression [5, 4]. Social impairments in ASD often persist across one’s development and across multiple domains, including communication, school, friendships, relationships, work, and community. Many individuals with ASD report difficulties making friends and navigating social norms and rate themselves as less socially competent compared to their typically developing peers [6]. Improved social skills are frequently an intervention target for individuals with ASD, with social skills training or social skills groups implemented in various formats [7]. Although social skills training (SST) is considered an evidence-based practice for school-age children with ASD, the evidence for effectiveness is variable [8] with some of the greatest challenges related to generalizing improved social skills outside of the therapeutic setting and fluently using skills across multiple settings, people, and situations [9]. This is not surprising, however, based on the complexity of understanding and demonstrating social skills and required fluidity to navigate the ever changing social norms and expectations across development and within the multiple domains described above (i.e., communication, school, work, etc.). Although the goal of SST is often to improve social competence for individuals with ASD, this is a lofty goal as social competence is a sophisticated construct encompassing numerous skills necessary to navigate these complexities. This is also likely reflected by the variable effectiveness and limited generalization. Bierman and Welsh [10] define social competence as the “social, emotional, cognitive skills and behaviors that children (people) need for successful social adaptation.” Their definition implies a broad range of skills and understanding, often not addressed in SST programs, including well developed emotional and cognitive skills.

Although research priorities in ASD now include a greater focus on adolescence and adulthood [11], less is known about interpersonal skills and social-emotional health for adolescents with ASD. In addition to biological changes associated with puberty, social norms and expectations become more complex during adolescence and reflect increased independence with less reliance on adults and more reliance on peers [12, 13]. Social interactions and friendships shift from play and turn taking towards a greater emphasis on conversation and building relationships [14]. Adolescents are also developing a sense of “self” with a heightened importance of healthy relationships and friendships in perceived self-worth [15]. For example, Bauminger and colleagues [13] found that friendship correlated positively with cognitive competencies and general self-worth and negatively with loneliness. Self-esteem and self-worth appear to be significant contributors to social-emotional health throughout adolescence [13] and negatively associated with symptoms of depression and anxiety [5]. Although there is an increased risk in general for emotional and behavioral problems into adolescence, females exhibit more internalizing symptoms such as anxiety and depression [16, 5] and often rate themselves lower on measures of self-esteem as compared to males [17].

Social competence is a crucial component of healthy functioning, especially during adolescence. This understanding emphasizes the importance of supporting individuals who have difficulties in the social domain in order to promote better overall functioning, especially social-emotional health. While it is clear that individuals with ASD are at increased risk for internalizing and externalizing disorders, we are still emerging in our understanding of how males and females with ASD may be affected differently and in relation to typically developing peers.

### Considering sex differences for adolescents with ASD

The available evidence suggests potential differences between males and females with ASD in terms of symptom expression at different points in their developmental trajectory [2]. For typically developing individuals, gender differences in social behaviors suggest female relationships are dyad-based, and interactions focus more on emotions and relationships [18] while male relationships focus more on shared interests or activities. Earlier studies on sex differences over the developmental trajectory for individuals with ASD suggested females with ASD are more cognitively impaired than males with ASD [19, 20], however some studies speculate higher functioning girls may be “missed” [4]. Kirkovski and colleagues [3] reviewed the available literature to examine gender differences in symptoms of ASD, demonstrating variability in findings across studies and the complexity of interpreting sex differences, or lack thereof, in the presentation of ASD. Some researchers suggest females may be more affected by their ASD during adolescence as compared to males [3, 5, 4, 14] and demonstrate greater social impairment during this time [21]. Solomon and colleagues [5] examined potential differences in girls and boys with high functioning ASD and also included typically developing comparison groups for both genders. Symptom profiles (language, social, repetitive behaviors) were similar for boys and girls with ASD, while girls with ASD differed significantly from typically developing girls in language and social abilities. Interestingly, girls with ASD demonstrated significantly more internalizing symptoms as compared to boys with ASD and typically developing girls. Solomon describes the increased risk of internalizing symptoms for adolescent girls and the increased risk of internalizing symptoms for individuals with ASD as a “double hit” (sex and diagnosis) for females with ASD [5].

### Social-emotional health for females with ASD

The relationship between social competence, self-perception, and levels of internalizing symptoms such as anxiety and depression are areas of focus in understanding social-emotional health across the developmental trajectory of ASD in boys and girls. This is especially important during the adolescent period when social differences between adolescents with and without ASD become more apparent and the risk for mental health problems increases. Females with ASD are of particular focus based on symptom presentation, including increased social impairments during adolescence [12, 13] and possibly their heightened risk of internalizing symptoms [5]. Biological changes associated with puberty result in more sophisticated adaptive skills required to maintain appropriate self-care, which is potentially problematic as some individuals with ASD demonstrate variable adaptive skills or skills significantly below what would be expected given their cognitive functioning [22]. Thus, for adolescent girls with ASD who have self-care skills below expected norms and limited social interactions, the potential impact on self-perception is significant. Although studies suggest sex differences in the characteristics of ASD [14, 2] and a substantial body of evidence identifies sex differences in social behavior [23, 18], studies rarely compare characteristics of females with and without ASD or consider these similarities and differences in their interpretation of the female autism phenotype. Due to this dearth of available literature targeting the specific experiences of adolescent females with ASD in comparison to their typically developing peers, little is known about the patterns of symptoms and inter-relationships among social competence, self-perceptions, quality of life, and problematic behaviors such as internalizing and externalizing symptoms for adolescent females with ASD in comparison to their typical peers. Cridland and colleagues [12] examined these experiences in a qualitative study through interviews with adolescent girls with ASD and their mothers. Although the sample was small, these early findings indicated that girls with ASD had challenges making friends, perhaps partially due to sustained involvement of mothers in self-care and social experiences, while noting that typical peers were increasingly independent in social experiences and self-care. Investigating potential relationships among social and emotional variables, including similarities and differences between adolescent girls with and without ASD, provides a platform for building hypotheses regarding potential protective and/or risk factors associated with social and emotional impairments. In this study, we examine specific research questions in working towards this effort.

#### Research questions

1. What is the relationship among indicators of social-emotional health (social competence, global self-worth, quality of life) for adolescent females with and without autism spectrum disorder?
  We hypothesize there is a strong relationship between social competence, self-perception, and quality of life for adolescent girls with autism spectrum disorder as well as for adolescent girls without ASD.
2. To what extent are there differences between adolescent females with and without autism spectrum disorder in measures of social-emotional health (social competence, social self-perception, quality of life) and problematic behaviors (internalizing and externalizing symptoms)?
  We hypothesize adolescent females without ASD will rate themselves higher than their peers with ASD in terms of social competence, self-perception, and quality of life and report lower rates of internalizing and externalizing symptoms.
3. How are problematic behaviors such as internalizing and externalizing symptoms related to the overall social-emotional health (social competence, global self-worth, quality of life) of adolescent females with and without ASD?
  We expect to find a significant, inverse relationship among problematic behaviors (internalizing and externalizing symptoms) and self-perception, among problematic behaviors and social competence, and among problematic behaviors and quality of life for adolescent girls with and without ASD. Further, we hypothesize that these inverse relationships will be stronger (especially for internalizing symptoms) in females with ASD because this group, by definition, has more difficulty with social interaction and as a result may experience more negative, internalizing processes than girls with less social difficulties.
4. How are social competence and problematic behaviors (internalizing and externalizing symptoms) related to parent perceptions of autism symptom severity?
  Given our statement above regarding the impact of social difficulties on internalizing symptoms, we would also hypothesize to find a significant relationship between autism symptom severity, ratings of social competence, and internalizing symptoms as reported by parents on the Social Skills Improvement System (SSIS). We expect that as parents rate autism symptoms as more severe, they would also report lower ratings of social competence and higher rates of internalizing symptoms.

## Methods

We explored the research questions above via a quantitative design utilizing correlational analysis and/or t-test comparisons. The independent variables included the existence (or not) of an autism spectrum disorder. Dependent variables included measures of social competence, self-perception, and quality of life as well as problem behaviors, specifically internalizing and externalizing symptoms. We examined data from an existing database within our lab containing data collected over the past 4 years.

### Participants

Tables 1 and 2 provide demographic characteristics for the sample, including sample sizes and mean scores for participants on measures of social-emotional health. Participants included adolescent females (14–19 years old) with and without ASD that participated in a social skills and self-care program for adolescent females, with participant data collection from six separate social groups over the course of a 4-year period (2010–2014). All participants lived in a large, Midwestern city or the surrounding suburbs. Inclusion criteria for females with ASD included (1) documentation of an ASD diagnosis by a psychologist, psychiatrist, developmental pediatrician, or interdisciplinary team (all diagnoses were based on DSM-IV-TR criteria), (2) reading skills at fourth grade level or higher, established by parent report or school evaluations when available, and (3) the ability to speak in 2–3 word phrases at a rate of 1–2 phrases per minute. Exclusion criteria (as part of the larger intervention program) included a recent history of significantly aggressive behavior suggesting the participant would be a danger to self or others. However, to date, no participants have been excluded from the study due to potentially dangerous behaviors. Participants in the ASD group were high functioning (no participants with a diagnosis of intellectual disability), with autism symptom severity scores ranging from 13–34, with mean scores in the above average range (mean = 20.48, SD = 4.91) on the SSIS. Participants demonstrated deficits in overall social skills as evidenced by the SSIS Social Skills composite scores falling in the below average range on the parent report (mean = 76.43, SD = 12.34) and in the lower end of average for self-report (mean = 89.16, SD = 13.93) measures. Female participants without ASD were peer mentor volunteers in the social skills program and deemed an appropriate volunteer for the program following a phone interview with the program director (i.e., commitment to attend weekly sessions, demonstrated appropriate behaviors, parental permission, and access to transportation). Although information regarding potential mental health diagnosis was not collected at screening, peer volunteers did not demonstrate any obvious social or mental health problems during the phone interview. Because data collection was part of a larger program evaluation examining the effectiveness of a social skills program for females with ASD, only females are included in this study.

**Table 1:** Demographic characteristics of GNO participants with and without ASD and parents of girls with ASD

Participant type	*N*	Mean age (SD)	% White
ASD	23	16.04 (1.72)	91 %
No ASD	29	16.75 (1.14)	97 %
Parents—ASD	23	n/a	n/a

Note: *n* size indicates maximum possible number that could have completed ratings. In many cases, the available *n* for selected analyses is smaller. Specific *n* sizes for analyses noted in text or separate tables. Age range of participants is 14–19. “% White” indicates the percentage of participants and parents who identified as white on demographic surveys completed as part of a larger program. All but two participants with ASD and one peer (no ASD) were of White descent. The two remaining participants were of Asian and Middle Eastern descent, and one peer was Hispanic

**Table 2:** Specific *n* sizes, mean scores, standard deviation, and range for global and subscale measures of social-emotional health, autism severity, and quality of life

Participant type	ASD		No ASD		Parents—ASD	
	*N*	Mean (SD)	*N*	Mean (SD)	*N*	Mean (SD)
SSIS—SS Composite	19	89.16 (13.93)	28	115.71 (11.26)	23	76.43 (12.34)
SSIS Communication	21	12.24 (3.67)	28	16.25 (1.92)	23	12.61 (3.03)
SSIS Cooperation	20	13.65 (2.48)	28	18.57 (2.03)	23	10.91 (2.88)
SSIS Empathy	21	12.33 (3.69)	28	15.93 (2.48)	23	10.65 (3.42)
SSIS Engagement	20	10.95 (4.16)	28	17.96 (3.10)	23	9.30 (3.50)
SSIS—PB Composite	21	117.29 (17.49)	28	87.71 (6.85)	23	132.91 (13.09)
SSIS Internalizing	20	15.25 (5.96)	28	2.54 (2.30)	23	14.52 (4.81)
SSIS Externalizing	21	11.81 (7.47)	28	3.29 (2.22)	23	11.43 (4.48)
SPPA—Global Self Worth	15	2.34 (0.83)	22	3.72 (0.32)	n/a	n/a
SSIS Autism Raw Score	n/a	n/a	n/a	n/a	23	20.48 (4.91)
YQOL Quality of Life—Gen	12	62.5 (28.78)	22	96.06 (8.58)	n/a	n/a
YQOL Quality of Life—Total	12	61.65 (16.0)	22	93.64 (8.57)	n/a	n/a

Note: Standard deviation and range presented in parentheses below *n* size for each measure and participant group. *SS* social skills, *PB* problem behavior

### Measures

Data collection included self and parent respondent measures to evaluate adolescent females’ perceptions of social competence as well as self-perceptions in various social-emotional domains. Data analyses in this study are based on measures completed at baseline prior to completing the social skills intervention program.

#### Social Skills Improvement System (SSIS)

The SSIS [24] is a standardized, informant-based rating tool designed to measure individuals’ social skills as well as problem behaviors. Respondents make ratings using a Likert-type scale on areas of social competence as well as social difficulty and challenging behaviors. Reliability is sufficient, with internal consistency coefficients in the upper 0.90s for Skills and Problem Behavior subscales. The SSIS has adequate test-retest reliability (parent form = 0.72–0.88; student form = 0.59–0.81) and validity and is correlated with other measures of social skills (r = 0.50–70). Analyses primarily include data from self-report forms. However, the parent report version provides data about the relationship between social-emotional health and parent perception of ASD severity.

#### Harter’s Self-Perception Profile for Adolescents (SPPA)

The SPPA [25] is a self-perception rating scale for adolescents between the ages of 14–18 years old. The 45-item instrument presents pairs of statements that describe adolescents and asks the individual to choose which statement from each pair best describes him/her and then to rate how much (a lot or a little bit) that statement describes him/her. Items come together to form subscales related to self-perceptions of global self-worth, as well as eight other domains. Internal consistency estimates range from 0.65 to 0.89. Using oblique rotation, factor analysis showed clear factor loadings for eight scales (0.47–0.84). The tool was developed in 1998 and updated in 2012 [26]. For this study, most participants completed the original (1988) version, with the most recent participants (*n* = 7 ASD, 6 no ASD) completing the updated edition.

#### Youth Quality of Life Instrument—Research Version (YQOL-R)

The YQOL-R [27] is a questionnaire completed by individuals between the ages of 12 and 18 years old. The 41-item instrument presents statements about an individual’s perceptions of self, relationships, environment, and general life enjoyment and satisfaction and asks the respondent to rate the degree to which the statement applies to them (10-point Likert-type scale ranging from “not at all” to “a great deal”). Items combine to form four subscales and a total scale score. Internal consistency estimates range from 0.77 to 0.96. Factor analysis showed a factor structure for four subscales and a principal component analysis provided support for a total scale (eigenvalue = 3.2).

### Procedures

#### Completion of measures

Upon being accepted to participate in the program, parents completed informed consent and participants under the age of 18 completed assents. Prior to starting the intervention, all participants and peers (no-ASD group) completed measures related to self-perceptions of social competence (SSIS) and self-worth (SPPA), along with quality of life (YQOL-R). Parents, primarily mothers (only 2 fathers served as the reporter), of individuals with ASD completed parallel parent forms of the SSIS. Parents of individuals without ASD did not complete parallel parent forms in an effort to minimize the paperwork burden for peer volunteer participants and their families. Only data collected prior to completing the intervention program are included in this study.

#### Data selection

Data for this study comes from a sample of 45 adolescent females that completed the aforementioned measures. Sample sizes for each outcome measure are presented in Table 2 by type of participant (no ASD and ASD). Sample sizes vary (*n* = 12–45) based on participant type (ASD vs no ASD), type of analysis, and availability and quality of outcome data. For example, in some analyses, we examined relationships among variables across collapsed groups, and for others, we parsed out the relationship for just individuals with ASD or no-ASD. Findings in this manuscript include participant data over the course of 4 years and across six intervention groups. Thus, variability of sample size is related to (1) changes in outcome measures employed across multiple years of program evaluation and (2) adequate completion of measures by parents and participants (i.e., missing or incomplete data).

### Data analysis

We calculated Pearson product moment correlations to examine relationships among adolescent self-ratings of social-emotional health (i.e., social competence, self-worth, and quality of life) as well as parent ratings of their daughter’s autism symptom severity, social competence, and problem behaviors. We conducted independent sample *t* tests (level of significance set at 0.05) to examine the differences between adolescent females with and without ASD on positive and negative indicators of social-emotional health. In addition to statistical significance, we evaluated the magnitude of differences or strength of relationships using guidelines established by Cohen [28]. For correlations, the r value will reflect a small (0.100), medium (0.243), or large (0.371) effect size. When comparing means, effect sizes will be interpreted as small (0.2), medium (0.5), and large (0.8).

## Results

### What is the relationship among indicators of social-emotional health (social competence, global self-worth, quality of life) for adolescent females with and without autism spectrum disorder?

First, we examined the overall relationship between global social competence (SSIS Composite), self-perception (SPPA Global Self Worth), and quality of life (YQOL-R Total QoL) across both groups. Results of the Pearson product moment correlation indicate a statistically significant (and large) relationship between global self-worth and global social competence for adolescent females, regardless of ASD diagnosis (*r* (36) = 0.74, *p* = 0.000). We also found large, significant relationships between social competence and quality of life (*r* (33) = 0.68, *p* = 0.000) and global self-worth and quality of life (*r* (32) = 0.72, *p* = 0.000) for the combined groups. Next, we examined the relationship between global social competence and global self-perception for the ASD and no-ASD groups, respectively. Correlations revealed a large and statistically significant relationship between social competence and global self-worth for the no-ASD group (*r* (22) = 0.62, *p* = 0.002) but not for the ASD group (*r* (14) = 0.32, *p* = 0.258). In evaluating the relationship between quality of life, social competence, and self-worth, this difference in effect was also noted between the ASD and no-ASD groups. For the no-ASD group, there was a large correlation between quality of life and social competence (*r* (22) = 0.81, *p* = 0.000) and for quality of life and self-worth (*r* (21) = 0.50, *p* = 0.021). However, there were not significant relationships between quality of life and social competence (*r* (11) = −0.177, *p* = 0.602) or for quality of life and self-worth (*r* (11) = −0.09, *p* = 0.801) for the ASD group.

**Fig. 1:**
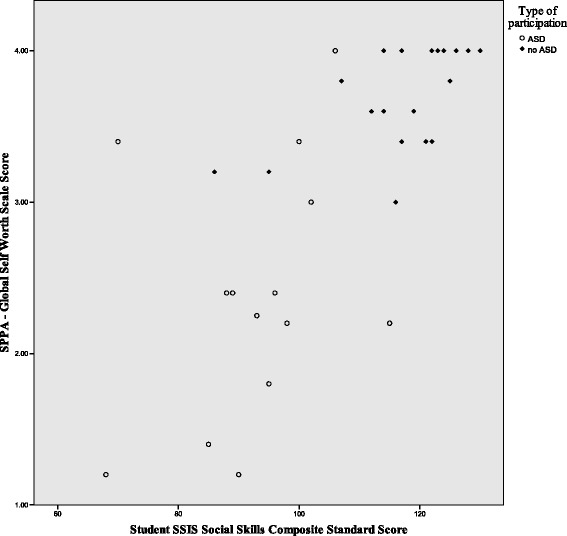
Scatter plot of self-worth and social competence self-ratings for ASD and no-ASD group

**Fig. 2:**
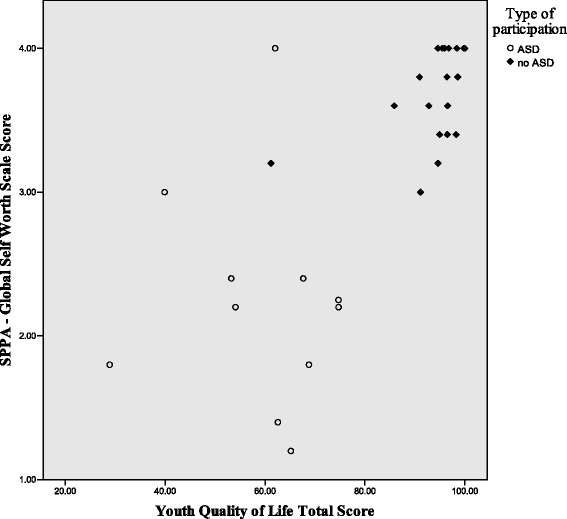
Scatter plot for self-ratings of self-worth and quality of life for adolescent females with and without ASD

**Fig. 3:**
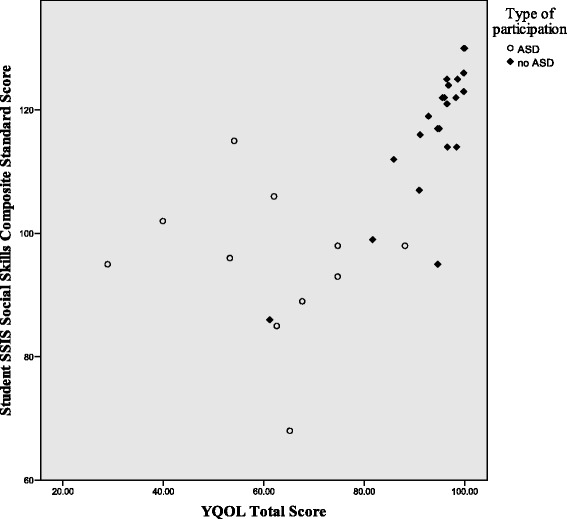
Scatter plot for self-ratings of quality of life and social competence, for adolescent females with and without ASD

Figures 1, 2, and 3 illustrate the differences in the patterns of relationship among quality of life, social competence, and self-worth between the ASD and no-ASD groups. For the no-ASD (peer) group, we note a truncated range of scores in the average to above average range, while for the ASD group, there is a wider dispersion of scores and these scores fall in the average to below average range. It is also important to note the differences in sample size for each analysis, with nearly double the number of scores available for the no-ASD group as opposed to those in the ASD group.

### To what extent are there differences between adolescent females with and without autism spectrum disorder in measures of social-emotional health (social competence, social self-perception, quality of life) and problematic behaviors (internalizing and externalizing symptoms)?

**Fig. 4:**
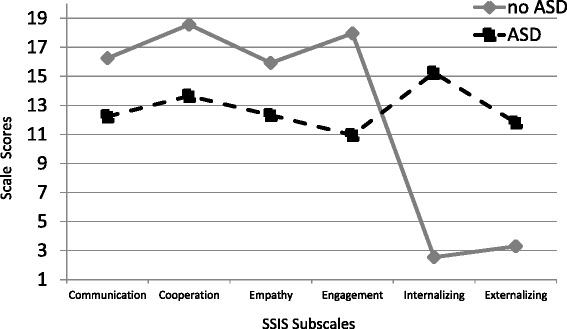
Selected subscales from the self-report version of the *Social Skills Improvement System* (*SSIS*). Figure depicts score differences between adolescent females with and without ASD. Higher scale scores indicate better developed skills. However, the Internalizing & Externalizing Subscales are reverse scored, thus higher scores reflect a greater level of concern. *Note*: Scores between 11-19 are considered in the Average range for Social Skills Composite subscales on the SSIS. For Problem Behavior Subscales of Internalizing and Externalizing Problems, scores between 1-14 are considered Average. Mean scores for the no-ASD group across SSIS subscales Communication, Cooperation, Empathy and Engagement ranged from 15.93-18.57 (SD = 1.92-3.10). Average scores for the ASD group ranged from 10.95-13.65 (SD = 2.48-4.16). For the reverse-scored items of the Internalizing and Externalizing subscales, means for the no-ASD group were 2.54 (SD=2.30) and 3.29 (SD= 2.88) for Internalizing and Externalizing respectively. For the ASD group Internalizing mean = 15.25 (SD =5.96) and for Externalizing mean = 11.81 (SD= 7.47)

**Fig. 5:**
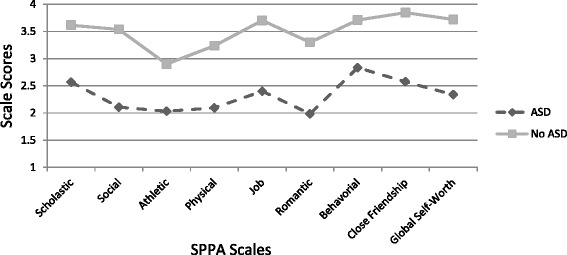
Differences between adolescent females with and without ASD in self-ratings on the Self-Perception Profile for Adolescents (SPPA) Global Self Worth and subscales. *Note*: SPPA scores range from 1-4, and scores between 2.25-2.75 are considered in the Average range. Mean scores across scales for ASD participants ranged from 1.98-2.57 (SD=.58-.84). Mean scores for no-ASD participants ranged from 2.90-.3.85 (SD=.32-.75). Effect Sizes ranged from 1.02-2.75

We conducted independent sample *t* tests to compare adolescent females with and without ASD on indicators of social-emotional health (social competence, self-perception, quality of life) and problematic behaviors (internalizing and externalizing symptoms). Results indicate statistically significant differences between groups on all measures with mean ratings for the ASD group lower (or worse) than the no-ASD group (see Table 2, Figs. 4 and 5). Females without ASD report higher social competence on the SSIS (*t* (45) = −7.21, *p* = 0.000, *d* = 2.11) and global self-worth on the SPPA (*t* (16.98) = −6.16, *p* = 0.000, *d* = 2.40) compared to females with ASD (see Table 2). As expected, females without ASD also reported a greater quality of life (YQOL-R Total Score) than those with ASD (*t* (14.25) = −6.44, *p* = 0.000, *d* = 2.60). Follow-up analyses indicated significant differences between the ASD and no-ASD group on all included subscales of both the SPPA and the SSIS and show large effect sizes (*d* = 1.02–3.08; see Fig. 4 for comparisons on the SSIS subscales and Fig. 5 for results of SPPA comparison). In terms of problematic behaviors, adolescent females with ASD reported significantly higher rates of internalizing symptoms (*t* (23.07) = −9.07, *p* = 0.000, *d* = 3.08) and externalizing symptoms (*t* (23.07) = −9.07, *p* = 0.000, *d* = 1.76) as compared to their no-ASD counterparts.

### How are problematic behaviors such as internalizing and externalizing symptoms related to the overall social-emotional health (social competence, global self-worth, quality of life) of adolescent females with and without ASD?

We first examined the overall relationships between problematic behaviors (internalizing and externalizing symptoms) and social-emotional health (social competence, quality of life, and global self-worth) collapsed across the ASD and no-ASD groups. Results show significant, and large, inverse relationships between internalizing symptoms and social competence (*r* (46) = −0.74, *p* = 0.000) as well as between internalizing symptoms and global self-worth (*r* (36) = −0.77, *p* = 0.000). We found a similar inverse relationship between internalizing symptoms and quality of life (*r* (33) = −0.69, *p* = 0.000). Thus, indicating that for both females with and without ASD, as higher internalizing symptoms were reported, girls reported lower ratings for social competence, self-worth, and quality of life. Results also show significant, inverse relationships across groups for externalizing symptoms and quality of life (*r* (34) = −0.47, *p* = 0.005), as well as social competence (*r* (47) = −0.76, *p* = 0.000) and self-worth (*r* (37) = −0.67, *p* = 0.000). Next, we examined these relationships within each group (ASD and no ASD). See Table 3 for a summary of all possible correlations. Results show significant, inverse relationships between problematic behaviors and social-emotional health for most analyses in the no-ASD group, with the exception of the relationship between externalizing symptoms—global self-worth and externalizing symptoms—quality of life. Results within the ASD group demonstrate small to moderate relationships between most constructs, with a significant correlation between the externalizing symptoms and social competence scales (*r* (19) = 0.59, *p* = 0.008) as well as between internalizing and externalizing symptoms (*r* (20) = 0.79, *p* = 0.000) and a modest correlation between internalizing symptoms and social competence (*r* (18) = −0.27, *p* = 0.284).

**Table 3:** Correlations among self-ratings of self-worth (SPPA Global), social competence (SSIS SS Composite), and externalizing (SSIS Externalizing subscale) and internalizing (SSIS Internalizing subscale) symptoms for adolescent females with and without ASD

	SPPA Global Self Worth		SSIS Social Skills Composite		SSIS Externalizing subscale		SSIS Internalizing subscale	
	ASD	No ASD	ASD	No ASD	ASD	No ASD	ASD	No ASD
SSIS Social Skills Composite	*r* = 0.32 p = 0.258 *n* = 14	*r = 0.62* *p = 0.002* *n = 22*	--	--	--	--	--	--
SSIS Externalizing subscale	*r* = −0.42 *p* = 0.117 *n* = 15	*r* = −0.30 *p* = 0.175 *n* = 22	*r = −0.59* *p = 0.008* *n = 19*	*r = −0.56* *p = 0.002* *n = 28*	--	--	--	--
SSIS Internalizing subscale	*r* = −0.41 *p* = 0.148 *n* = 14	*r = −0.43* *p = 0.048* *n = 22*	*r* = −0.27 *p* = 0.284 *n* = 18	*r = −0.49* *p = 0.008* *n = 28*	*r = 0.79* *p = 0.000* *n = 20*	*r = 0.72* *p = 0.000* *n = 28*	--	--
YQOL-R Total QOL	*r* = −0.09 *p* = 0.801 *n* = 11	*r = 0.50* *p = 0.021* *n = 21*	*r* = −0.18 *p* = 0.602 *n* = 11	*r = 0.81* *p = 0.000* *n = 22*	*r* = 0.26 *p* = 0.417 *n* = 12	*r* = −0.39 *p* = 0.069 *n* = 22	*r* = 0.06 *p* = 0.864 *n* = 11	*r* = −0.59 *p* = 0.004 *n* = 22

Note: italic values indicate significance at *p* ≤ 0.05

Differences in effect were found for the patterns of relations between internalizing symptoms and social competence, self-worth, and quality of life. For all three relationships, the effect for the no-ASD group was significant but not for the ASD group. The relationship between internalizing symptoms-self-worth was moderate and inverse for both the ASD (*r* (14) = −0.41, *p* = 0.148) and no-ASD groups (*r* (22) = −0.43, *p* = 0.048). For internalizing social competence, the ASD group effect (*r* (18) = −0.27, *p* = 0.284) was smaller than the no-ASD group (*r* (28) = −0.49, *p* = 0.008). And for internalizing quality of life, there was an inverse, moderate effect for the no-ASD group (*r* (22) = −0.59, *p* = 0.004), but no effect for the ASD group (*r* (11) = 0.06, *p* = 0.864).

**Fig. 6:**
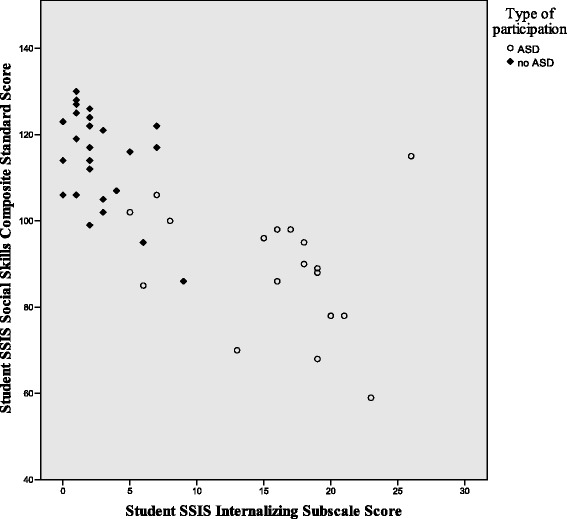
Scatter plot of self-ratings of internalizing symptoms and social competence, for adolescent females with and without ASD

**Fig. 7:**
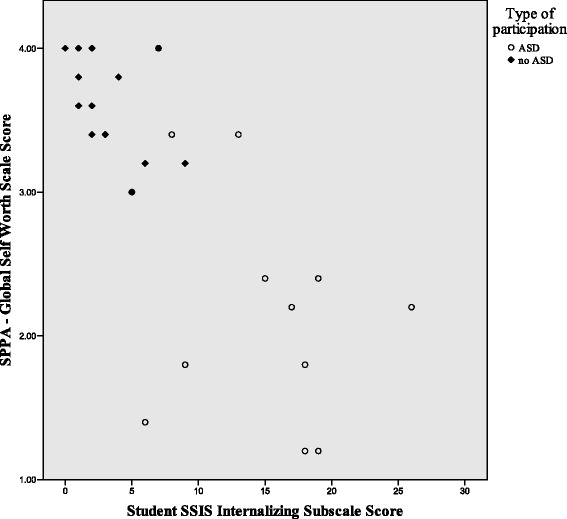
Scatter plot of self-ratings for internalizing symptoms and global self-worth for adolescent females with and without ASD

**Fig. 8:**
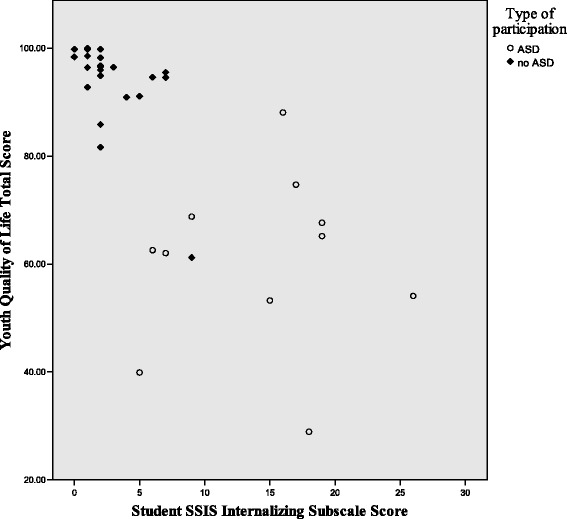
Scatter plot of self-ratings of internalizing symptoms and quality of life for adolescent females with and without ASD

To further examine the pattern of relationships, specifically the differences in effect between groups for the relationships between internalizing symptoms and social-emotional health, grouped scatter plots are shown in Figs. 6, 7, and 8. While the scatter plots illustrate the strong overall inverse relationship, for both groups, there is a truncated range of scores for the no-ASD group, lessening the strength of relationship between variables within respective groups. It also illustrates the significant differences in the relationships between internalizing symptoms and both social competence and self-perceptions between the girls with and without ASD. Scatter plots show a cluster of ratings in the average to above average range for peer participants, and a greater spread of scores in the below average lower range for participants with ASD. Additionally, their ranges do not overlap.

### How are social competence and problematic behaviors (internalizing and externalizing symptoms) related to parent perceptions of autism symptom severity?

Autism symptom severity is indicated by the Autism Symptom Index on the SSIS parent report form. We used parent ratings in these analyses because this index is not included on the self-report form. We first examined the relationship between student and parent ratings on the SSIS, in order to examine consistency of perceptions of social competence and problem behaviors between parents and daughters. Because analyses revealed nonsignificant relationships between parent and daughter ratings for global social competence, global problem behaviors, and internalizing symptoms, only parent ratings were used for this analysis. This inconsistency between parent and daughter report is not unique to individuals with autism but is a phenomenon seen in adolescent-parent reports on the whole and is near that reported in the SSIS manual [24]. Results from the Pearson correlation show that there was not a significant relationship between parent ratings of their daughter’s autism symptom severity and their rating of her internalizing symptoms (*r* (23) = 0.20, *p* = 0.354) or externalizing symptoms (*r* (23) = 0.29, *p* = 0.176). There was however a significant, inverse relationship between autism symptom severity and social competence, as measured by the SSIS Autism Symptom Index and Social Skills Composite (*r* (23) = −0.61, *p* = 0.002). Thus, as parents rated their daughter’s autism symptoms as more severe, they made lower ratings of social competence. We examined this effect more closely by evaluating the correlation between the Autism Symptom Index and specific Social Skills and Problem Behavior subscales on the SSIS (summarized in Table 4). Follow-up correlations indicated significant, inverse relationships between autism symptom severity and the specific social skill domains of communication, empathy, and engagement (*r* = −0.57 to −0.72) but not for cooperation (*r* (23) = −0.15, *p* = 0.499). Correlations among problem behavior subscales of internalizing and externalizing symptoms and autism symptom severity were not significant (see Table 4).

**Table 4:** Correlations among parent ratings of autism symptoms and their perceptions of their daughter’s social competence on the SSIS

	Autism Raw Score—SSIS
SSIS Social Skills Composite	*−0.61*
	*p = 0.002*
SSIS Communication	*−0.72*
	*p = 0.000*
SSIS Cooperation	−0.15
	*p* = 0.499
SSIS Engagement	*−0.66*
	*p = 0.001*
SSIS Empathy	*−0.57*
	*p = 0.005*
SSIS Problem Behavior Composite	0.36
	*p* = 0.091
SSIS Internalizing	0.20
	*p* = 0.354
SSIS Externalizing	0.29
	*p* = 0.176

Note: Only parents of participants with ASD completed SSIS (*n* = 23)

Italic values indicate significance at *p* < 0.01

## Discussion

We examined existing data to explore the relationship between selected indicators of social-emotional health and problematic behaviors specific to internalizing and externalizing symptoms for adolescent females with and without ASD. We compared self-perceptions of social competence, self-worth, and quality of life between groups and examined the impact of internalizing and externalizing symptoms on these constructs. Lastly, we examined the impact of autism symptom severity as rated by parents (according to the SSIS) on social competence and problem behaviors.

Overall, there was a significant relationship among the constructs of social-emotional health (quality of life, social competence, global self-worth), across both ASD and no-ASD groups. This relationship remains for the no-ASD group, however, for females with ASD in this study, the relationship among social competence, self-worth, and quality of life diminishes to small or nonexistent. Adolescent females with ASD rated themselves significantly lower in all three areas compared to their non-ASD peers. Social impairments, such as in ASD, are reflected in the lower ratings of social competence made by girls with ASD. Adolescence includes changes in social norms and expectations that result in complex interactions and relationships with a greater emphasis on independence and less reliance on adults. Research indicates sex differences in social behaviors throughout development, with female friendships possibly more complex, including a greater emphasis on emotions and relationships [18]. Biological changes associated with puberty impact self-care routines and related adaptive behaviors, which could prove even more difficult for some individuals with ASD who demonstrate variable or delayed adaptive behavior skills [22]. Harter and colleagues [25] found that self-perceptions about appearance and friendships were most related to self-esteem and that relationships significantly contribute to global self-worth. Our findings, showing that females with ASD made significantly lower ratings in these areas, along with poor reported social competence and quality of life, suggests the potential for a significant impact on their social-emotional health. Consistent with some recent literature [5, 29], females with ASD in our sample also reported more internalizing and externalizing symptoms as compared to typically developing females. In general, females experience more internalizing symptoms as compared to males [30]. In our sample, we found an extremely large difference between adolescent females with and without ASD, suggesting internalizing symptoms significantly impact the female ASD population. Thus, if adolescent females with ASD have lower self-perceptions, report lower quality of life, and report poor social competence in addition to greater problem behaviors compared to peers, they become an extremely vulnerable population with significant risk to develop co-existing mental health conditions and related problems. Although only significant for the no-ASD group, we found moderate correlations (*r* = 0.41–0.43) between internalizing symptoms and global self-worth. The increased risk for problematic internalizing and externalizing symptoms, coupled with complex social relationships with social impairment as a core feature in ASD, results in girls with ASD experiencing a “double whammy” in adolescence or “double hit” as described by Solomon [5]. We also described this “double whammy” with respect to sex and diagnosis, suggesting that a unique intervention program is needed to address the complex needs of adolescent females with ASD [31]. While all adolescents experience an increased complexity of social norms and expectations and biological changes associated with puberty, females with ASD experience both the potential impact of ASD (social impairments, difficulties in adaptive behavior) as well as sex (focus on emotion, conversation, relationships; increased risk for internalizing symptoms; and biological changes that significantly impact self-care routines). This “double whammy,” along with additional variables that contribute to unique challenges for this population (e.g., primarily male peer group, research based on male samples, limited focus on adolescents and adults), supports the need to target the social-emotional health in this population.

Interestingly, we found significant relationships between indicators of social-emotional health and problematic behaviors across groups but no longer found significance within the ASD group when parsing out the relationships among these variables within each group. As noted above, correlations between internalizing symptoms and global self-worth were moderate and similar for both groups with a lack of significance in the ASD group as a result of a smaller sample. As seen in the scatter plots (Figs. 3, 4, 5, 6, 7, and 8), ratings for adolescent females without ASD generally indicated better social-emotional functioning and fewer problematic behaviors compared to those in the ASD group and fell within a truncated, clustered range (average to above average). Ratings for adolescent females with ASD generally indicated lower functioning and were more dispersed in terms of score ranges (average to extremely low). Larger samples are needed to determine if the pattern of data would suggest similar general relationships within both groups or if data would reveal a significant relationship in constructs within only one group.

In an attempt to determine how ASD symptom severity is related to social-emotional health, we examined the relationship between autism symptom severity (according to parent report on the SSIS), social competence, and problematic behaviors (internalizing and externalizing symptoms). There were no significant relationships between parent ratings of ASD symptom severity and their ratings of internalizing or externalizing symptoms. In contrast, there was a significant, inverse relationship between parent ratings of autism symptom severity and their perceptions of their daughter’s social competence. As parents perceived a higher intensity and severity level of their daughter’s autism symptoms, they also saw a negative impact on her social competence. For adolescent females with autism, this finding supports the notion that parents continue to be aware of the impact of their daughter’s symptoms and see it continue to affect her social relationships in significant ways. Parent data for typically developing females was not collected, thus, a comparison of similarities and differences was not able to be conducted in the same manner. Some research, including data from our recent qualitative research, indicates that parents continue to be involved at a high level in their daughters’ social experiences, which is in contrast to the reduction in parent involvement in social activities and increasing social independence of typically developing adolescents [12]. A larger sample is needed to further explore the relationship between these variables and how autism symptom severity may or may not impact social-emotional health for adolescent females with ASD. Understanding these relationships could lead to hypotheses about directional causation and interventions that target and promote possible “protective factors” (e.g., autism symptom severity, self-perception) resulting in improved health and less impairments during adolescence.

Exploring the interplay between social-emotional health and problematic behaviors such as internalizing and externalizing symptoms in the context of autism is a crucial first step to better understand the experiences of adolescent females with ASD. These findings further demonstrate how girls in adolescence experience an intersection of social complexity and sense of self—where social experiences and self-concept relate to one another and affect one’s quality of life, which in turn influence one’s ability to address internal stress and inhibit challenging behaviors. Our data indicate that young women with ASD see themselves as more challenged, reporting lower levels of social competence, self-concept, quality of life, and higher rates of negative internal and external symptoms, meaning they are at higher risk for negative experiences across the domains examined in this study. Parent reports also elaborate the impact of autism on the functioning of these young women, indicating that for young women with ASD, as their symptoms are more elevated, their social competence is lowered. The impact of autism on a population that is already at higher risk due to increased social complexity, higher risk for negative self-concept and internalizing disorders, supports the need for prevention and intervention tailored to the unique experiences of this population.

## Conclusions

We acknowledge some limitations in this study. Although sample size is relatively small and varies between groups and for specific measures, results suggest robust effects and an adequate sample size for most analyses. Females represent less than 15 % of participants in published autism studies, with a very small proportion of research (approximately 1 %) focusing specifically on females with ASD [32], suggesting studies with small samples could make a significant contribution to the literature. Findings should be interpreted and generalized within the context of our limited sample. Participants were primarily white, living in suburban areas. Participants in the ASD group are mostly representative of higher functioning females with ASD, although not validated through cognitive testing for inclusion in the study. Our sample of females without ASD might represent a somewhat “biased” sample of typically developing adolescent females. There could be something inherently different about this group, as all participants agreed to volunteer as peer participants for a social skills program. However, the use of only baseline data limits potential bias from intervention effects and could mirror similar biases likely to occur for similar situations in which participants volunteer for research. Parent report measures are not available for participants in the no-ASD group, limiting data analysis to comparisons between self-report measures and examining relationships in parent report data only in the ASD group. Findings are also limited by only including respondent measures and a lack of multiple measures evaluating each construct. Additionally, this study focused only on differences between females and did not include comparisons between males with and without ASD. As this study was preliminary and emerged from a broader program evaluation of a social skills curriculum designed for females, males were not included. Future studies comparing males and females with and without ASD would provide a much broader picture of social-emotional health in adolescents and would allow for an analysis of sex-specific similarities and differences in social-emotional constructs and how the expression of ASD symptoms impacts males and females. Although lack of a male comparison group is a limitation in understanding potential sex differences, the inclusion of a female, typically developing reference group is a strength of the study and contributes to the purpose of this study which is to better understand females with ASD in comparison to their peers.

Further analyses are needed, with larger data sets, comparisons with adolescent males, and measures of autism symptom severity from the perspective of the adolescent and other sources. These analyses could also inform a broader model of the degree to which various factors of the experience of adolescent females with ASD (autism symptoms, internalizing disorders, and negative self-perceptions) could contribute to their social competence and vice versa. Understanding these relationships ultimately informs intervention programming to more effectively support skill development that promotes healthy social-emotional development and protective factors to address the risk of internalizing symptoms and greater impairment during adolescence.

These findings have implications for supports and intervention programming. Understanding the relationship among indicators of social-emotional health and problematic behaviors speaks to the importance of developing specialized programming that addresses not only skills crucial for building social competence but that also involves developing positive self-concept and thus potentially protecting against internalizing symptoms. In terms of intervention, a program such as the *Girls Night Out* model [31] incorporates curricula related to social competence and self-care (both in terms of physical hygiene and positive self-concept), delivered through evidence-based strategies within the natural environment. This model (*GNO*) targets specific skills related to the “double whammy” described earlier and is designed to promote social-emotional health across the domains explored in this study and address a critical need for this vulnerable population. Future research and intervention programs should consider potential sex differences in social behavior as well as individual differences related to the expression of ASD when examining social-emotional constructs and generalizing findings.

## Abbreviations

ASD: autism spectrum disorder
SPPA: Self-Perception Profile for Adolescents
SSIS: Social Skills Improvement System
YQOL-R: Youth Quality of Life Instrument—Research Version
SST: social skills training
